# Novel Iodine nanoparticles target vascular mimicry in intracerebral triple negative human MDA-MB-231 breast tumors

**DOI:** 10.1038/s41598-020-80862-5

**Published:** 2021-01-13

**Authors:** Sharif M. Ridwan, James F. Hainfeld, Vanessa Ross, Yaroslav Stanishevskiy, Henry M. Smilowitz

**Affiliations:** 1 Department of Cell Biology, University of Connecticut Health Center, 263 Farmington Avenue, Farmington, CT 06030 USA; 2 Nanoprobes, Inc., 95 Horseblock Road, Yaphank, NY 11980 USA

**Keywords:** Breast cancer, Cancer, CNS cancer, Experimental models of disease, Translational research

## Abstract

Triple negative breast cancer (TNBC), ~ 10–20% of diagnosed breast cancers, metastasizes to brain, lungs, liver. Iodine nanoparticle (INP) radioenhancers specifically localize to human TNBC MDA-MB-231 tumors growing in mouse brains after iv injection, significantly extending survival of mice after radiation therapy (RT). A prominent rim of INP contrast (MicroCT) previously seen in subcutaneous tumors but not intracerebral gliomas, provide calculated X-ray dose-enhancements up to > eightfold. Here, MDA-MB-231-cells, INPs, CD31 were examined by fluorescence confocal microscopy. Most INP staining co-localized with CD31 in the tumor center and periphery. Greatest INP/CD31 staining was in the tumor periphery, the region of increased MicroCT contrast. Tumor cells are seen to line irregularly-shaped spaces (ISS) with INP, CD31 staining very close to or on the tumor cell surface and PAS stain on their boundary and may represent a unique form of CD31-expressing vascular mimicry in intracerebral 231-tumors. INP/CD31 co-staining is also seen around ISS formed around tumor cells migrating on CD31^+^ blood-vessels. The significant radiation dose enhancement to the prolific collagen I containing, INP-binding ISS found throughout the tumor but concentrated in the tumor rim, may contribute significantly to the life extensions observed after INP-RT; VM could represent a new drug/NP, particularly INP, tumor-homing target.

## Introduction

Triple Negative Breast Cancer (TNBC) accounts for 10–20% of diagnosed breast cancers. At the time of diagnosis, TNBC are more likely to have already metastasized^1,2^. Common sites of metastases are the brain, lungs and liver. A challenge of treating these metastases is addressing tumor proliferation while limiting the dose of treatments, such as radiation, to normal tissues. Despite recent advances in chemo-, immune-, and radiation therapy for breast to brain metastases, outcomes are still measured largely in months. While optimizing the use of stereotactic and whole brain radiation therapies can extend life and limit toxicity to some extent, conceptually new approaches are needed to help these patients. One such new approach that we pioneered is high atomic number (Z) nanoparticle enhanced radiation therapy pioneered by Nanoprobes, Inc. and the Smilowitz lab, first published in 2004^3^. Gold Nanoparticles (AuNPs) and more recently Iodine Nanoparticles (INPs) have been shown to increase the specificity and dose of radiotherapy by enhancing the local dose in tumor regions^3–9^. Other high-Z nanoparticles have also been studied, including those of gadolinium^10^, hafnium^11^, holmium^12^, bismuth^13^, platinum^14^, iron^15,16^. Largely due to the photoelectric effect, electrons are ejected from the K and L shells after X-ray capture; these electrons travel a relatively short distance and deposit their energy locally in the tumor^4^. Previous studies using advanced experimental gliomas in mice showed that both AuNPs and INPs localize to the tumor region after intravenous injection^6,8,9,17^ and significantly extended median survival of mice with advanced orthotopic gliomas by more than twofold^6,8^. Microlocalization studies performed on mouse brains bearing advanced human U87 gliomas showed the INPs to be largely localized to the tumor endothelium^8^ suggesting that the resulting increased radiation dose may cause greater damage to the tumor endothelium^18^. Consistent with this finding was the observation that INP-enhanced RT (INP-RT) synergized with DOXIL chemotherapy greatly increasing the efficacy of chemotherapy to treat an advanced glioma in mice^8^. In a recent study using microCT we showed that the INPs also localize to advanced human triple negative breast cancers growing in the brains of mice where they concentrate to very high levels in a ~ 100-micron rim of the tumor^9^. The efficacy of RT (15 Gy RT at the Stony Brook RT100 facility, given as a single radio-surgical dose) was also greatly enhanced by the presence of the INPs; median survival was increased by ~ 2.6-fold; remarkably, four out of 10 mice experienced ≥ fivefold life extensions with 3 of the mice (30%) experiencing > tenfold life extensions. Single dose 15 Gy irradiations lead to tumor shrinkage in these mice^9^ unlike that which has been observed with advanced U87 gliomas^8^.

In this immunofluorescence study we show that the INPs are found throughout the tumor but are especially concentrated in a ~ 100-micron rim of the tumor leading edge that corresponds to the rim of high contrast seen by MicroCT, due to high iodine concentration. Like the U87 glioma, most of the INP staining in this intracerebral 231 model co-localizes with CD31 stain and is found on or very close to the tumor cell surface. The INP + CD31 staining tumor surface forms the boundaries of irregularly-shaped structures (ISS) that might be a form of vascular mimicry (VM)^19–22^ i.e. the ability of cancer cells to organize themselves into vascular-like structures used to provide nutrients and oxygen independently of normal blood vessel angiogenesis or vasculogenesis^23^. Collagen I (Col I) co-localizes with the INPs on the ISS/VM boundary; since INPs interact with Col I directly, Col I is likely to be one of the sites on the ISS/VM to which the INPs bind. We hypothesize that it is the very high dose of radiation to these structures and adjacent radiation sensitive targets that is responsible for the extraordinary tumor control we observe^9^. We believe we are the first to identify NP targeting to ISS/VM structures. ISS/VM could represent an INP/nanoparticle/drug-homing therapeutic target and that INP-enhanced RT would likely benefit patients with breast cancer-brain metastases since a correlation between vascular mimicry and poor outcome has been made^24^.

## Materials and methods

### Institutional animal assurance

Animal experiments were conducted according to NIH guidelines and approved by the University of Connecticut Health Center Institutional Animal Care and Use Committee (IACUC) before start of the study.

### Tumor cells, tumor model and mice

MDA-MB-231 human triple negative breast cancer cells, obtained from the ATCC were transduced with the pFULT vector, under the control of the human PGK1 promoter to express both luciferase and tomato, by the Skin Biology and Disease Resource-Based Center at Northwestern University, Chicago, Il. Athymic nude mice purchased from Charles River were used. Fifty to one-hundred thousand luciferase and tomato expressing MDA-MB-231 cells were implanted 2.5–3.0 mm deep into the striatum of nude mice through a 0.5 mm diameter burr hole made on the left coronal suture of the skull 2/3 of the way between the midline and the temporalis muscle insertion^25^.

### Iodine nanoparticles (INPs)

INPs consist of polymerized triiodobenzene with a PEG coating. Their synthesis is detailed in Hainfeld et al.^7^.

### Treatment/irradiation

Tumors were grown to an advanced stage, reaching 5 × 10^8^–10^9^ photon counts assessed by IVIS. The group treated with INPs at 7.0 g I/kg were IV injected with a concentration of 70 mg I/ml INPs (4 injections, 0.5 ml each, 2 each day spaced > 3 h apart). Irradiations were not used for this study but were utilized for a recently published companion study^9^. Briefly, 24 h after the last injection, mice were irradiated with 15 Gy 100 kVp X-rays. Mice were anesthetized with 140 mg/kg ketamine and 3 mg/kg xylazine in phosphate buffered saline given intraperitoneally in about 0.06 mL. The head and body were protected by a 3.4 mm-thick lead shield with a notch that enabled irradiation 8.0 mm caudally from the posterior canthus of the left eyelid and dorsally from the dome of the palate to above the calvarium. Irradiations used a Philips RT100 X-ray generator (Amsterdam, The Netherlands) operating at 100 kVp with a 1.7 mm Al filter. Dose was calibrated using a Radcal ion chamber (Monrovia, CA). To prevent lethal brain edema, dexamethasone (5 mg/kg) was injected subcutaneously 18 and again 6 h before irradiation and 6 and again 18 h after irradiation^26^.

### Histology, immunostaining and immunofluorescence

Twenty-four hours after an IV injection of 7 gI/kg INPs, mice were cardiac perfused and fixed. Brains were removed, cryopreserved and cryosectioned. Cryopreserved brain tissues were cut into 7 μm-thick coronal sections using a Cryostat (Leica, Cat. #: CM 3050S) at ~ -24–26 °C. DAPI 1:1000 (4,6-diamidino-2-phenylindole) was used to stain nuclei. Primary antibodies used were rabbit anti-PEG (1:500; Abcam Cat # AB512572) and goat anti-CD31 (1:100; Abcam Cat # 19,808) goat and anti-Collagen I (1:200; Millipore Cat # AB758). Secondary Antibodies used were donkey anti-rabbit Alexa Fluor 488 (1:400; Invitrogen A21206), and donkey anti-goat Alexa Fluor 647 (1:200; Life Technologies A21447). Tissue sections were incubated overnight in primary antibodies. After washing four times with PBS, secondary antibodies were added for 8 h. After washing four times and coverslip mounting, imaging was performed using a widefield fluorescence microscope (Zeiss Axio Observer Z1) and a confocal fluorescence microscope using 10 × and 63 × objectives (Zeiss LSM 880). The coverslips were then floated off and the same sections were stained, both in our laboratory and by Histowiz, Inc. with PAS with Hematoxylin counterstain^27–29^.

### Dot blots

Collagen I (Gibco A1048301) was applied to a nitrocellulose membrane (BioRad 1,620,146) at the indicated concentrations as a 0.5ul spot (strips ABCD). After drying, membrane strips were blocked with 5% BSA in TBS-T for 1 h. After a final TBS-T rinse, A, B, C strips were incubated with 2.5 mg/ml INP solution for 1 h, followed by TBS-T washes on a rocker (3 × 5 min). A, B, C, D strips again were blocked with 5%BSA in TBS-T for 30 min followed by a final TBS-T rinse. Then, A, D were incubated with Rabbit anti-PEG antibody (1:500; Abcam catalog # AB512572) in 2% BSA in TBS-T for 30 min. The membranes were then washed with TBS-T (3 × 5 min) and A, B, D were incubated with Goat anti-Rabbit-IgG-HRP (1:2000; Abcam, Catalog# ab205718) in 2% BSA in TBS-T for 30 min. The membranes were then washed with TBS-T (3 × 5 min) followed by a milli-Q deionized water wash (3 X 5 min). Silver staining was then performed using the Enzmet^tm^ HRP detection Kit (Nanoprobes Inc., Catalog# 6001–30 ml) following the manufacturer’s instructions.

## Results

**Figure 1:**
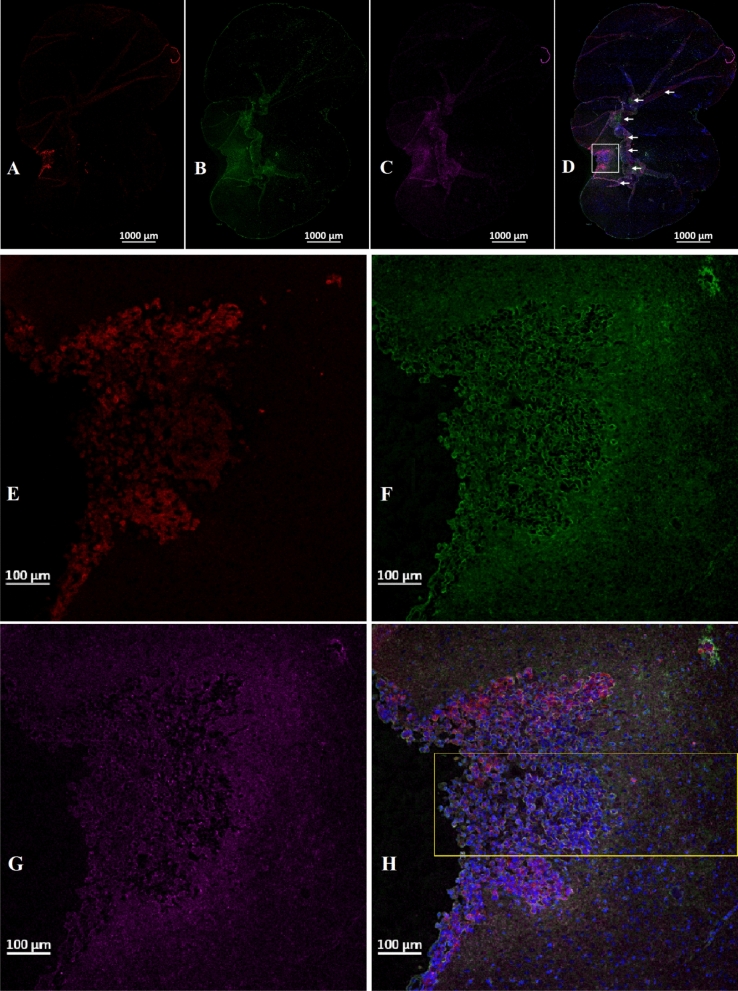
Confocal images (10 ×) of brain coronal sections (**A**–**H**) with advanced MBD-MD-231 tumors 24 h after iv INP injections. **A**–**D**, 10 × images of tumor residing in left-hemisphere; E–H, tumor and peritumor region in white square in **D** is magnified in (**E**–**H**). Nuclei (dapi = blue); **A**, **E**, Tumor cells (td-Tomato, red); **B**, **F**, INPs (anti-PEG, green); **C**, **G**, CD31 (antiCD31, violet); **D**, **H**. (all four channels). White arrows, some artefactual folds that formed during the preparation of the section. INPs are seen to be taken up mostly in the tumor and peritumor region where they mostly colocalize with CD3, nominally an endothelial stain.

**Figure 2:**
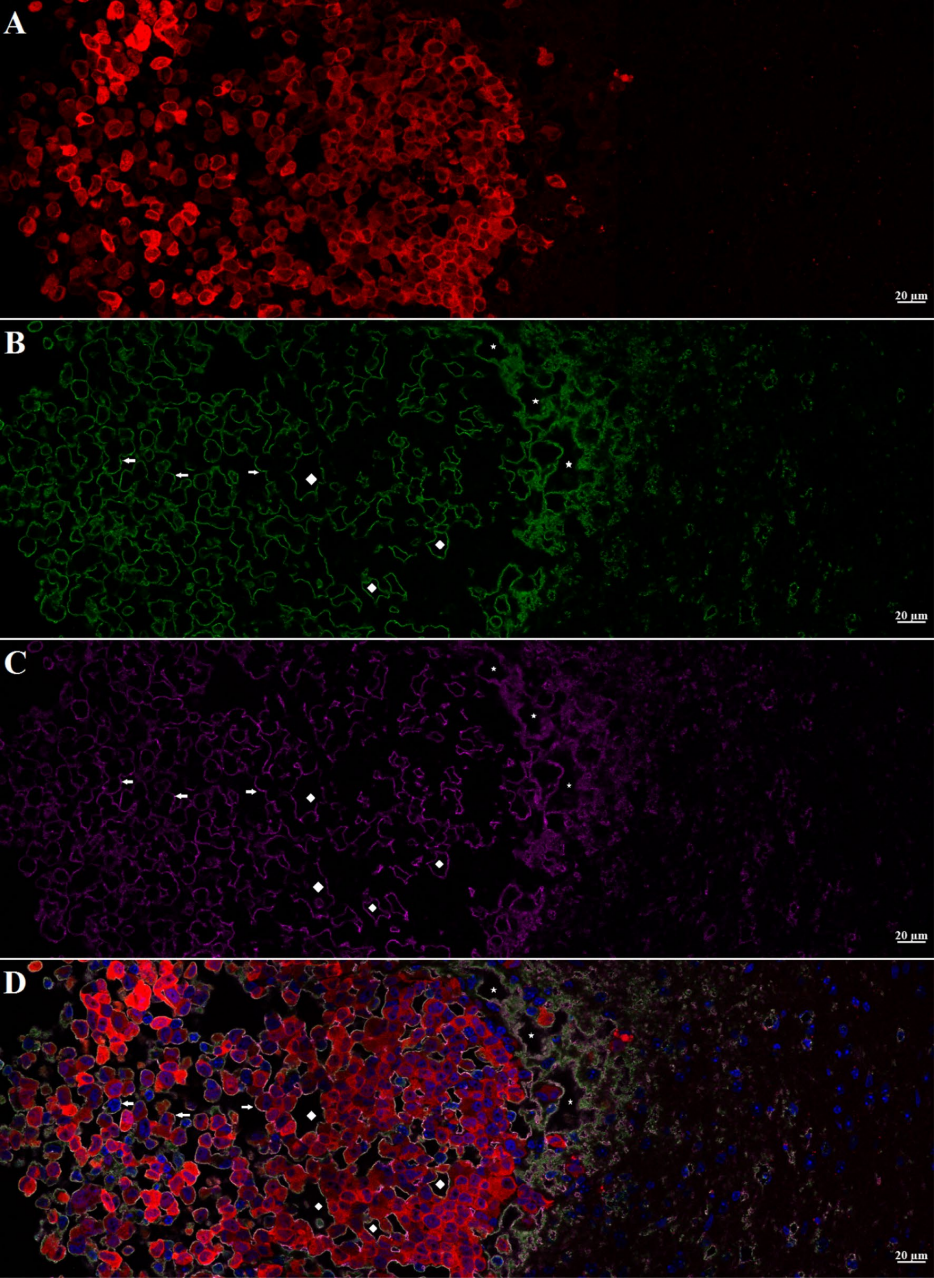
Higher magnification (63 ×) Confocal images of yellow boxed area shown in Fig. 1H. **A**–**D**: showing MBD-MD-231 tumor, tumor edge and peritumor area. **A**, tumor cells (td-Tomato, red); INPs (Anti PEG, green); Endothelial Marker (Anti-CD31, violet), **D**, all four colors merged with nuclei stained with DAPI, blue. Highest INP staining intensity colocalized with CD31 at the tumor edge and peritumor region compared to the tumor center. Arrows: Groups of tumor cells with grape cluster appearance. Irregularly shaped spaces (ISS) delineated by endothelial stains (asterisks and diamonds). Diamonds: Spaces formed are contributed by tumor cells only Asterisks: Spaces formed are contributed by both tumor and non-tumor cells.

**Figure 3:**
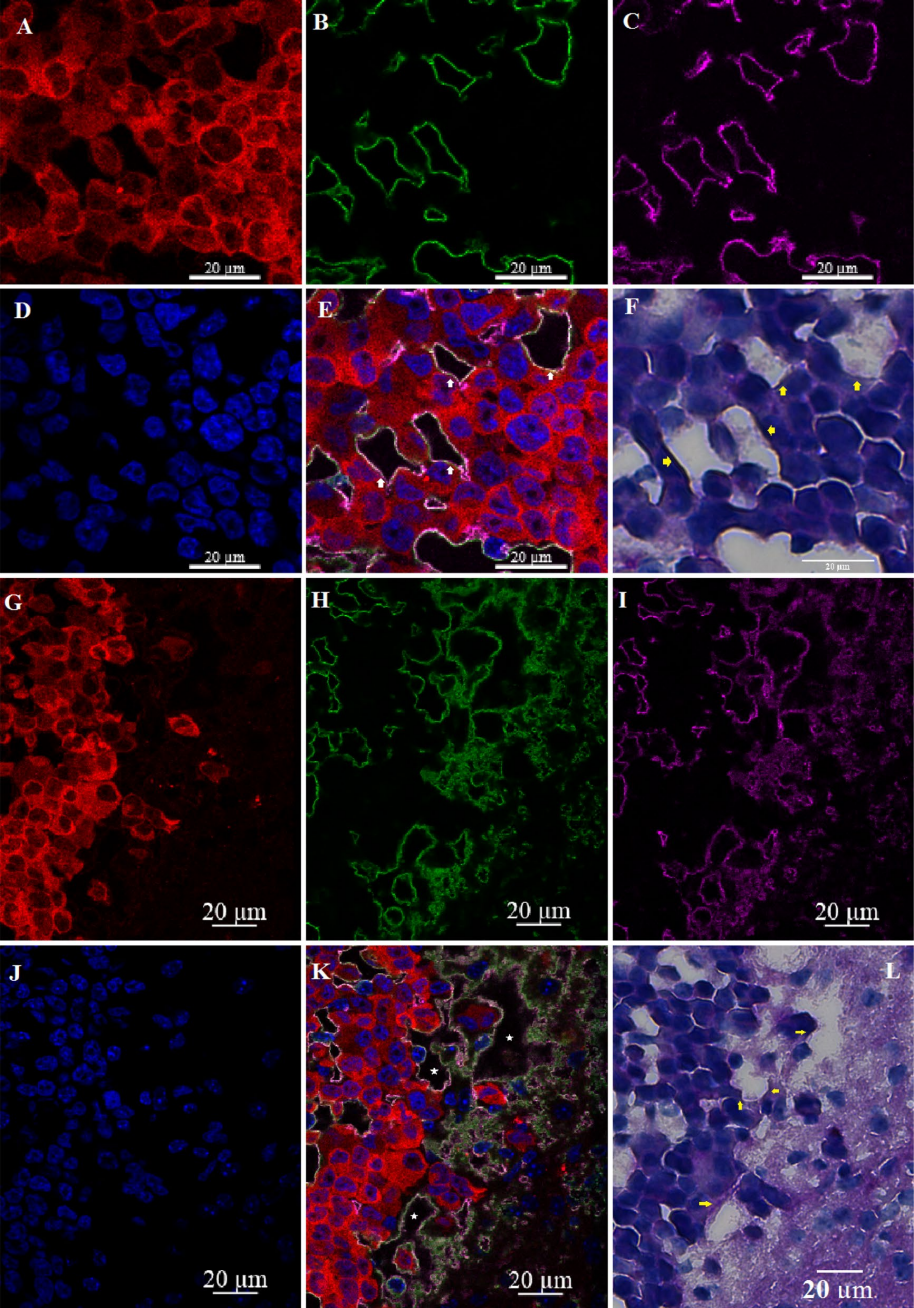
Irregularly shaped spaces (ISS) in the tumor (**A**–**F**) and at the tumor edge or active zone (**G**–**L**). **A**, **G**, Tumor cells (td-Tomato, red); **B**, **H**, INPs (antiPEG, green); **C**, **I**, Endothelial Stain (antiCD31, violet); **E**, **K**, All colors combined with Nuclei stained (Dapi, Blue); **F**, **L** (PAS stain). Yellow Arrows: Darkened PAS stain just outside of ISS. ISS (white arrows and asterisks) have all the hallmarks of vascular mimicry but are also bounded by CD31 stain.

**Figure 4:**
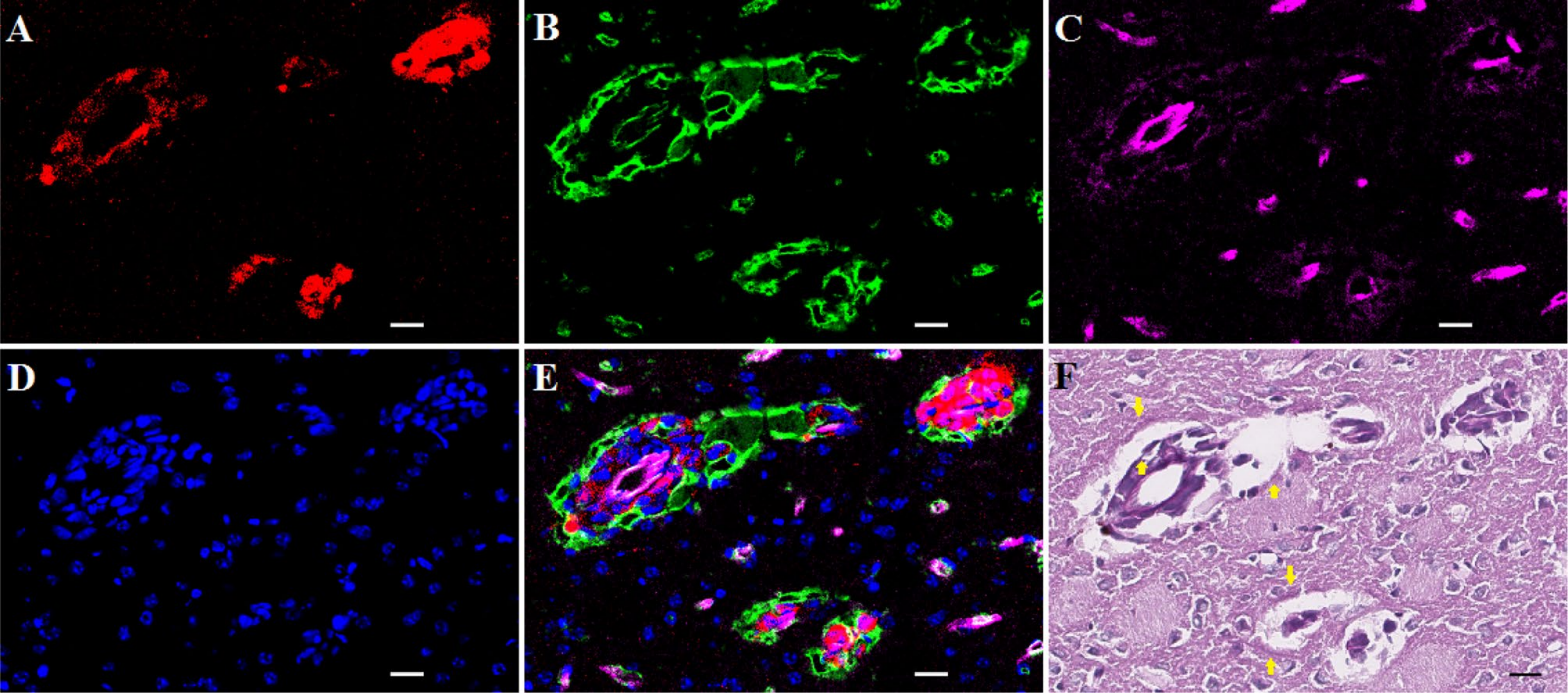
Migrated tumor showing ISS with INP and CD31 association. White and black bars = 10 um. **A**, Tumor cells (td-Tomato, red); **B**, INPs (antiPEG, green); **C**, Endothelial stain (antiCD31, violet); **D**, Nuclei (Dapi, Blue); **E**, Colors combined; **F**: PAS stain. Arrows in 4F: Hue of darkened PAS stain immediately outside INP and CD31 stained ISS forming around migrated tumor cells.

**Figure 5:**
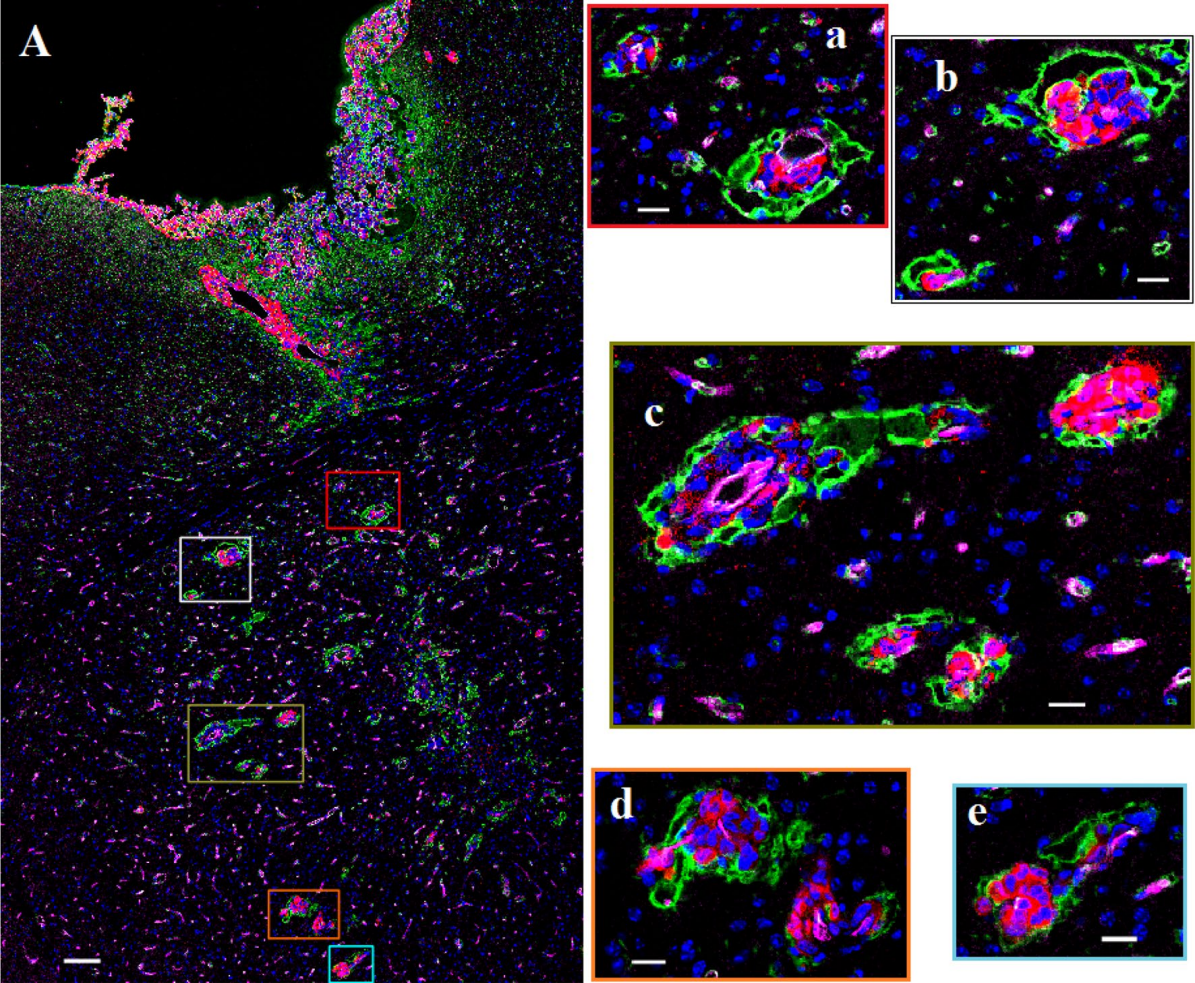
Fluorescence labeled confocal images (20 ×) of left cerebral hemisphere of athymic nude mice bearing MBD-MD-231 tumors 24 h after iv INP administration. **A**. Main tumor mass with migrated tumor loci deep into the cerebrum with INP uptake seen as a bright ring. White bar = 100 um. **a**–**e**: enlarged fluorescent image of the migrated tumor foci; white bar = 20 um. Tumor cells (td-Tomato, red); INPs (anti-PEG, green); Endothelial stain (antiCD31, violet); Nuclei (Dapi-Blue). Images are of all four colors combined. Intense CD31 stain is seen around blood vessels with associated tumor-cells. Green INP staining does not permit the lower intensity antiCD31 stain to show through.

**Figure 6:**
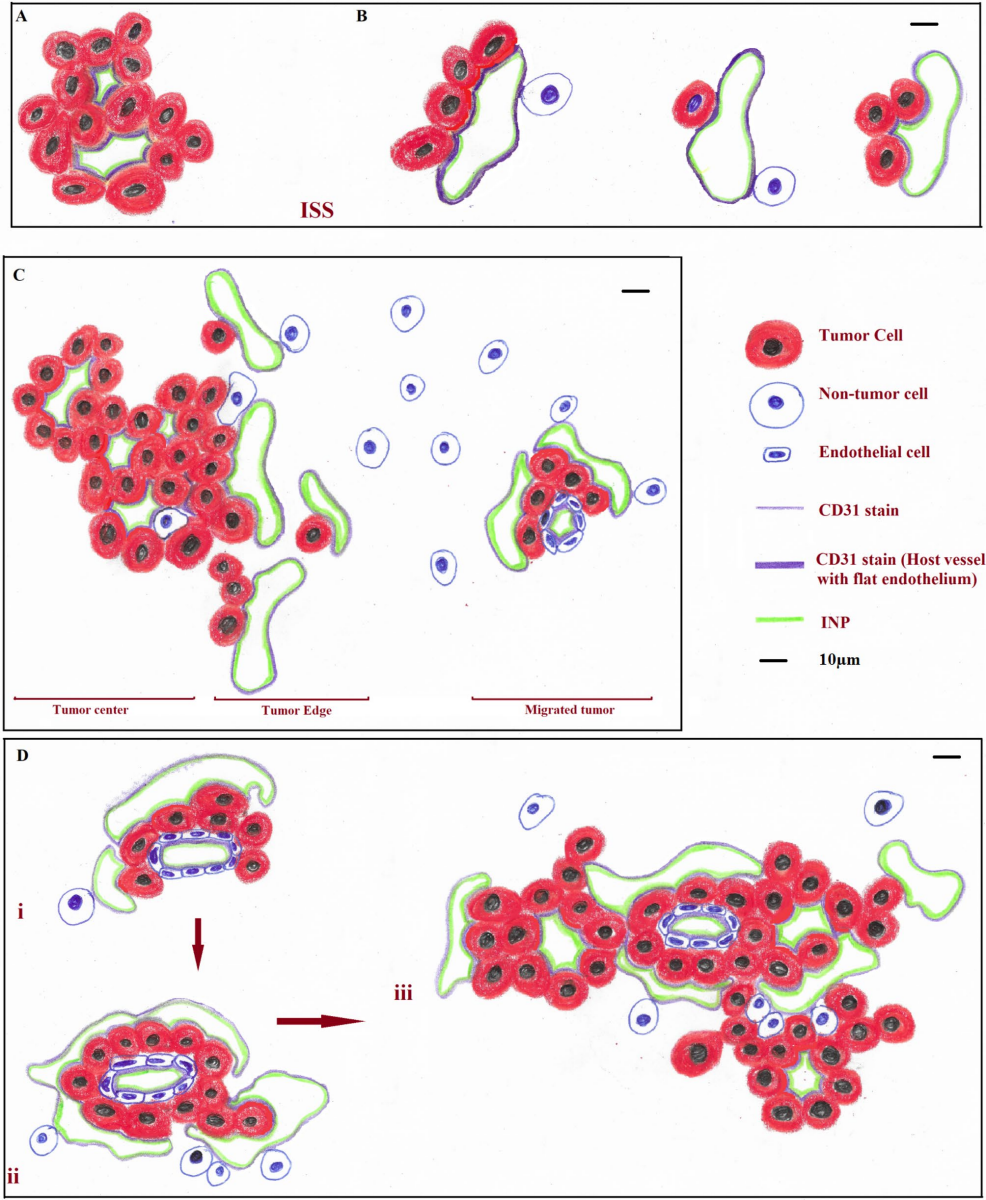
Diagrammatic summary of the relationships between MBD-MB-231 tumor cells, I-SS (vascular mimicry) and INP and CD31 labeling in the inner tumor, the tumor rim and tumor cells that have migrated along blood vessels. **A**. ISS bounded by tumor cells only; **B**. ISS bounded by tumor cells partially; **C**. ISS in the main tumor center, tumor edge and in at the migrated tumor; **D**. Proposed process of formation and maturation of ISS. INP and CD31 representations are intended to be colocalized.

**Figure 7:**
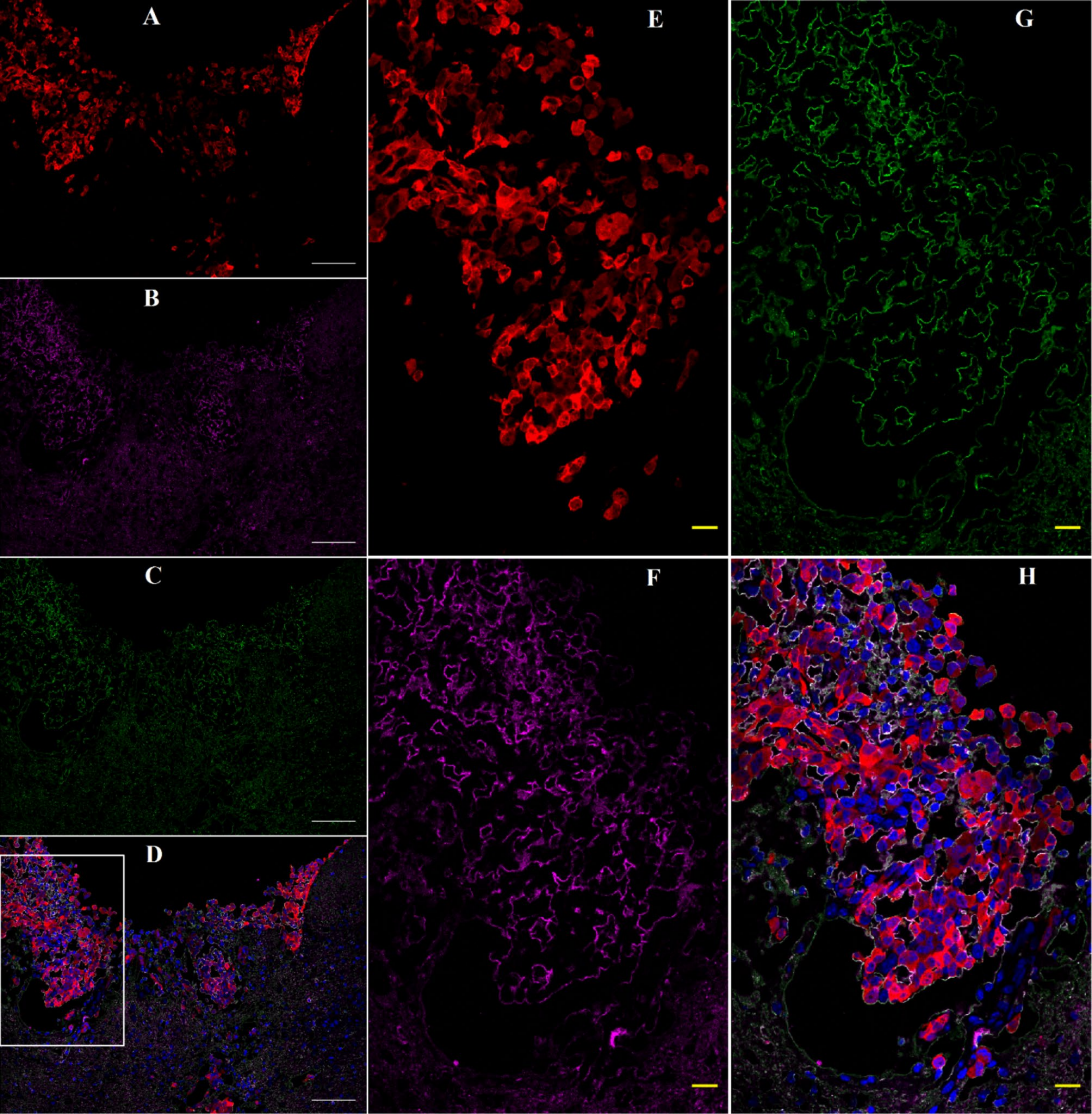
Confocal images (63 ×) of mouse brain with an advanced 231TL tumor 24 h after iv INP injection: Colocalization of INPs and Collagen I. Immunofluorescence was performed as described in Methods. Panels **A**–**D** are low power images; **E**–**H** higher power images of the boxed region in panel **D**. tDTomato = tumor cells; Green = INP; Magenta = Collagen 1; Blue = Dapi (Nuclei). White inset in figure D is enlarged in E–H. White bar = 100 µm; Yellow bar = 20 µm.

**Figure 8:**
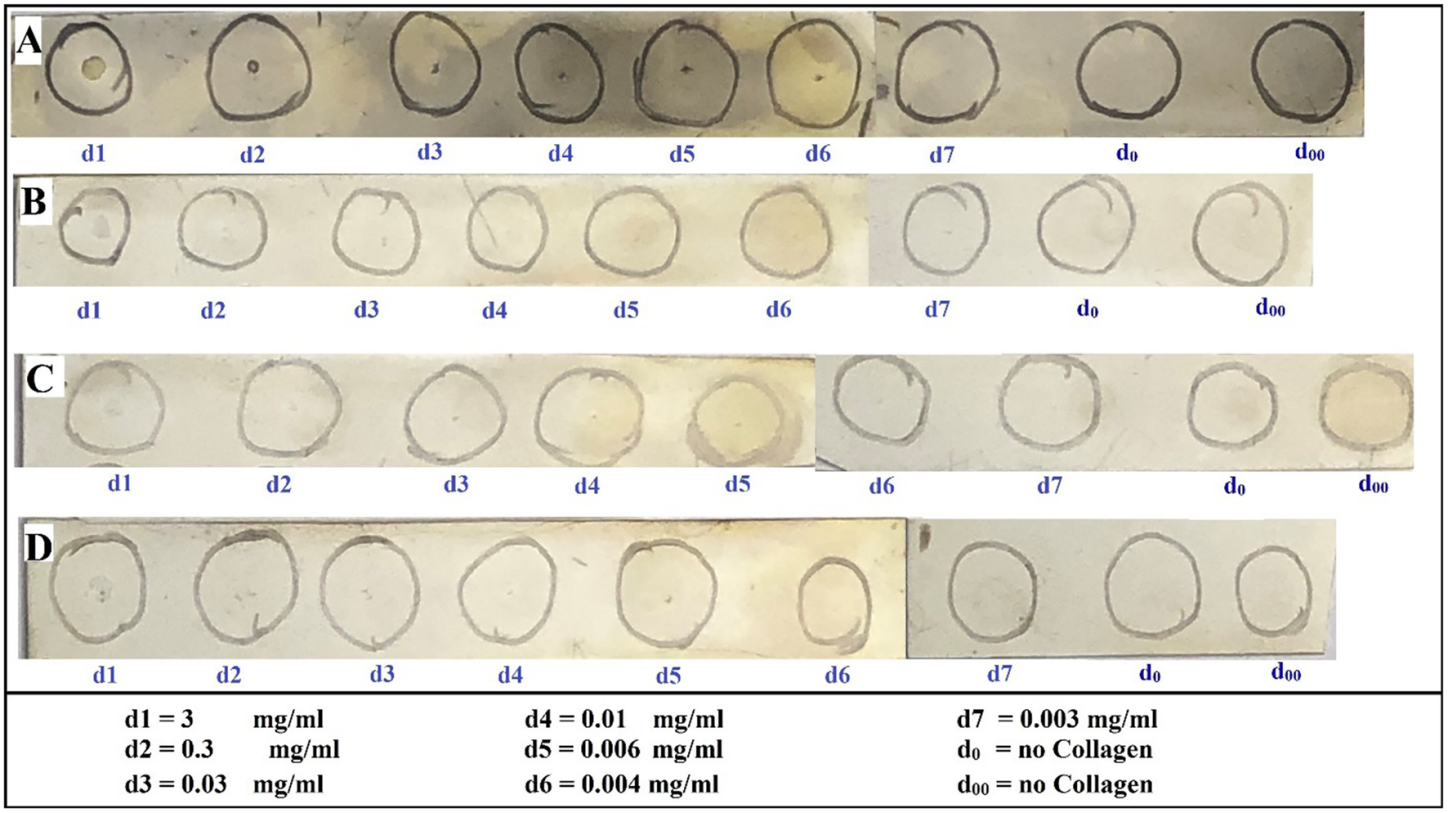
Dot-blot showing positive interaction of INP with Collagen-1: **A**–**D**: Collagen-1 (0.5ul of solutions at indicated concentrations d1–d7) was added to the center of each circle. **A**–**C**, Incubated with INPs (2.5 mg/ml); **D**, Not incubated with INP. **A**, **D**, Both primary and secondary antibodies; **B**, Secondary antibody only; **C**, No antibody control.

INP micro-localization studies may provide a clue as to the mechanisms by which the INPs prolong life when combined with RT. Athymic nude mice bearing advanced intracerebral MDA-MB-231 tumors expressing both luciferase and td-Tomato (red fluorescent protein) were injected with 7 g iodine/kg INP iv and cardiac-perfused fixed 24 or 72 h after the last injection. Cryosections (7 microns) were stained for the presence of INPs (Anti-PEG) or the endothelial marker CD31 (Anti-CD31). All sections were DAPI stained for nuclei. Figure 1A-D shows low power (10 ×) confocal images. Arrows point to where the tissue folded on itself creating staining artefacts. Nevertheless, it is clear that INPs are localized to the region of the left hemisphere containing the tumor (seen as red fluorescence) and peri-tumor region. CD31 staining is more prominent there as well. The region shown in the white box is shown in Fig. 1E-H. These enlarged 10 × images clearly show the INPs that are localized to the tumor region. A swath of more intense green fluorescence (50–100 u meter wide) is seen on the edge of the tumor (Fig. 1F). A band of increased CD31 fluorescence is also seen (Fig. 1G). INP and Anti-CD31 fluorescence appear to co-localize (cf. Fig. 1F,G). The rectangular box (Fig. 1H where tomato, INP, tumor and nuclear fluorescence images are merged) is examined at 63 × (Fig. 2A-D). Tumor cells appear red (Fig. 2A). Migrating tumor cells are seen on the tumor edge. INP fluorescence (Green) is shown in Fig. 2B. The most intense fluorescence is seen at the tumor edge where INP labeling appears to be predominantly around irregularly-shaped spaces (ISS) that also are labeled by anti-CD31 (Fig. 2C). Consequently, a higher density of CD31 labelling is also seen at the tumor edge. In the interior of the tumor, arrows highlight small clusters of tumor cells (Fig. 2B-D). Careful inspection shows that these grape-like clusters of tumor cells typically are surrounded by CD31 and INP staining that form the boundaries of the ISS. Asterisks denote ISS outlined by CD-31 and INP stain– and invariably some degree of tumor stain. These structures are even more prevalent in the tumor periphery. Figures 3A-F feature ISS in the tumor center; Figs. 3G-L focus on the tumor periphery where INP and CD31 staining is most intense. PAS immunohistochemical staining, used previously to help identify vascular mimicry^27–30^ has been added. In the tumor center, INP and CD31 are colocalized largely to the surface of tumor cells that surround the ISS. Many of these spaces stain positively for PAS stain, seen as dark staining along the tumor edge (Arrows, Fig. 3F and Supplementary Fig. 1). INP and CD31 fluorescence was rarely seen on inter-tumor cell to cell boundaries. In the tumor periphery, more intense INP and more abundant CD31 fluorescence is seen. ISS co-labeled with INP and CD31 abound. The ISS are not necessarily bounded by tumor cells on all sides but all are bounded by tumor cells to some degree. It appears that ISS are found wherever tumor cells have migrated. While INP and CD31 stains are found all around the perimeter of the ISS that have formed around migrated tumor foci, CD31 staining is less intense. Figure 4A-F show tumor cells that have migrated away from the main tumor mass, deeper into the normal brain. Even very small numbers of tumor cells result in a ISS – with its rim of intense INP and less intense CD31 fluorescence. ISS surrounding the tumor cell cluster are also positively stained with PAS. Figure 5A shows a main tumor mass with tumor cells that have migrated into the brain. Surrounding each of the migrated tumor cell clusters are ISS that are labeled with INPs and CD31 stain. Supplementary Fig. 2 shows migrated cells and cell clusters 72 h after INP injections. An extensive network of co-localized INP and CD31 stain is seen in every case. INP and CD31 staining is specific; background staining in non-tumor containing regions of the left and right hemispheres show very little, if any INP and CD31 staining as shown in Supplementary Fig. 3. Negative staining controls in which either first, second or both antibodies are omitted showed virtually no staining (data not shown). Supplementary Figs. 4 and 5 are additional images showing migrating tumor cells with ISS and attendant INP and CD31 staining. PAS staining showed darkened PAS stain adjacent to ISS. Figure 6 is a diagram depicting the variety of INP and CD31 staining structures (ISS) we see in the tumor center, tumor periphery and around migrating tumor cells. The sequence and mechanisms by which the ISS develop and 3-dimensionally interconnect are yet to be deciphered. Figure 7 shows colocalization between CD31 and Collagen I (Col I) using a primary antibody to Col I, and immunofluorescence. Figure 7 panels A-D are low power images; E–H higher power images of the boxed region in panel D. Non-tumor regions (Supplementary Fig. 6 Panel B) showed no positive staining for Col I. Controls omitting the primary antibody (Supplementary Fig. 6 panel C) and no antibody controls (Supplementary Fig. 6 panel D) were devoid of immunofluorescence. Attempts to establish the presence of Col I in normal mouse breast lactation ducts were inconclusive due to background staining and will take more experimentation to establish or refute. Antibodies to Col III, Col IV, fibronectin and vitronectin showed no immunofluorescence staining (data not shown). Dot blots revealed specific INP-Col I binding as shown in Fig. 8. The data suggest that INPs interact with Col I that is localized to CD31 containing ISS*/*VM boundaries that are found throughout the MDA-MB-231 tumor growing in the mouse brain but are especially plentiful at the tumor rim.

## Discussion

Basic information about the novel iodine nanoparticle used has been reported recently^7^. Briefly it is a crosslinked triiodobenzene polymer with a PEG coating, having a hydrodynamic diameter of about 20 nm, a blood half-life of 40 h, and shows no obvious toxicity after an IV dose of 4–7 g iodine/kgbw. INP treated mice showed the same weight gain as age-matched controls, CBC and blood chemistry tests were normal, and histopathology of major organs showed no signs of inflammation or fibrosis.

In a recent study^9^, we reported a second tumor type growing in the mouse brain for which the intravenously injected INPs provide a clear-cut therapeutic benefit following radiation therapy—the human MDA-MB-231 triple negative breast tumor—(referred to as 231). Since iv injected INPs enhance RT of both the advanced 231 and U87 gliomas growing in the brains of mice, the possibility is raised that INP-enhanced RT may be effective to treat many different intracerebral tumors including lung tumors, the most common type of tumors to metastasize to the brain. MicroCT studies of mouse brains implanted with 231 cells that were allowed to become advanced showed that, like gliomas, the INPs localize to the regions of the brain containing the tumor. Indeed, ~ 20:1 ratios of tumor to non-tumor ratios were found using both the U87 and 231 models. However, the tumoral distribution of the INPs differed from what was found for intracerebral U87 tumors. Unlike the U87 glioma where the INPs are found distributed in a more or less heterogeneously homogeneous fashion^8^, very bright contrast was found along the rim of the 231 tumors^9^, reminiscent of what we have seen for mice with subcutaneous breast tumors after AuNP injections^31^. While INP contrast is distributed throughout intracerebral U87 human gliomas^8^, INPs accumulated to a higher extent in the rim of the 231 tumors^9^. INP levels in the rim of the 231 tumors were calculated to give dose enhancements of > fivefold 24 h after INP injections and up to eightfold in rim-regions 72 h after INP injections. Such enormous dose enhancements might be expected to provide significant benefit to the mice receiving RT. While median life extensions of ~ twofold were seen for both the intracerebral U87 and 231 models, 40% of the 231 mice showed substantially longer survival. Survival was > fivefold for one mouse and > tenfold for 30% of the other mice. IVIS data suggested that tumors all but disappeared before returning nearly a year later. We hypothesize that the extremely high levels of INPs found in the rim of the tumor are contributing to the extraordinary efficacy we observe. It is therefore important to decipher the mechanisms by which the INPs provide such extraordinary life extensions.

In this paper we have examined the microscopic distribution of the INPs using confocal fluorescent microscopy. We have shown that INP staining is also greatly increased in the intracerebral MDA-MB-231 tumor rim. This observation not only corroborates the MicroCT data showing increased iodine in the tumor rim, but supports the contention that anti-PEG antibody labels the INPs; importantly, anti-PEG staining in the absence of INP injection is negative. Anti-CD31 fluorescence is also greatest in the 231-tumor rim and is co-localized with INP fluorescence. The high levels of INP in the tumor rim are predicted to provide large radiation dose enhancements that are largely confined to the structures to which the INPs are bound due to the very short distance the ejected electrons are predicted to travel^32^. Therefore, the structures to which the INPs bind may represent an important target for therapy of intracerebral metastases. What are those structures? Our data suggest that the 231 tumor cells, whether they be packed in the tumor center or the tumor periphery (rim) or migrating away from the tumor to initiate new foci of tumor growth, participate in the formation of irregularly shaped structures (ISS). In the tumor center, ISS appear to be bounded by clusters of only tumor cells as we do not observe cell nuclei around the ISS that do not express red fluorescent protein. INPs and CD31 fluorescence are found all around the perimeter of the ISS—either on the tumor cell surface or on a structure very close to the tumor cell surface. The ISS show positive PAS staining on their border. Based on the literature^27–30^ we hypothesize that ISS bounded only by tumor cells represent VM—a subset of VM that has CD31 staining. Previously most accounts report the absence of CD31 associated with VM^27–29^ with the exception of B16 melanoma^19^. It is possible that 231 brain tumors express CD31 in the brain, but not when growing subcutaneously; this can be easily tested. One possibility is that some of the CD31 associated ISS we see may also be related to the abnormal breast duct formation reported in a CD31 transfected breast tumor study^33^ raising questions about the possible relationship between the tendency to form ducts and VM. At the tumor rim, ISS are found in greater abundance. They are invariably found associated with red-fluorescent tumor cells. However, tumor cells are not necessarily found on all sides of the ISS. Nevertheless, the ISS are bounded by INPs and CD31 on all sides suggesting that INP and CD31 binding may not be interacting with the tumor cell surface but to a distinct molecular-entity. INP staining is particularly abundant in the tumor rim. A similar situation exists when analyzing tumor cells that have migrated a distance from the tumor edge—usually along a blood vessel as evidenced by bright CD31 staining and flattened nuclei (Supplementary Fig. 2B,C). What might the INPs and CD31 be interacting with?

Previously we showed that gold nanoparticles (AuNPs) appear to localize to extracellular matrix in bladder tumors in mice^34^. While the AuNPs and INPs have very different compositions, they are both ~ 20 nm and they both have PEG on them. One possibility is that the PEG on the INPs are binding to one or more connective tissue components that may be produced by the tumor cell and possibly other cells in the tumor environment and deposited along the boundary of the ISS—such as basement membrane component(s). Our findings support this hypothesis. We find the boundary of the ISS to contain collagen I (Col I)—but not Col III, Col IV, fibronectin or vitronectin. What is the significance of Collagen I in the tumor distribution of NPs in general, and INPs? **a**. We have shown that INPs bind to Collagen I on dot blots (Fig. 8), identifying a novel NP binding site localized to the tumor. Since PEG has been shown to interact with Col I to form hydrogels^35^, it is likely that PEG mediates the interaction of INPs with Col I; future studies could test this hypothesis. Proteomic analyses are needed to establish that INPs bind to Col I in vivo and to identify other possible INP binding sites in addition to Col I. Collagen I was predominantly found in tumors (Fig. 7 and Supplementary Fig. 6A) and does not appear to be expressed in normal brain (Supplementary Figs. 6B) therefore providing an explanation for the largely tumor-specific INP localization/binding we have shown, **b**. Collagen I is usually found in extracellular matrix and possibly basement membrane like structures. Our histologic study shows Collagen I localized either to the tumor cell membrane itself or a structure very close to or adjacent to the tumor cell surface. Further studies could identify the cellular origin of the Collagen I and the exact structure to which it is associated, **c**. Since endothelial containing vessels appear to be in low abundance relative to ISS/VM, we suggest that the INPs may get into the ISS/VM system from the blood and bind to Collagen I. This finding has significant implication for NP drug delivery to tumors with extensive ISS/VM since the endothelial barrier appears to be largely absent enabling direct delivery of NPs possibly bypassing the blood brain barrier. **d**. Enhanced drug delivery to tumors using NP targeted to ISS/VM could represent a significant method to increase drug delivery to brain tumors and addresses an unmet clinical need, **e**. Our previously published report^8^ demonstrated that INP binding (shown in this paper to be to the collagen I-containing structures lining the ISS/VM) greatly enhanced RT efficacy.

The ISS may then represent a new target for intracerebral brain tumor therapy; the INPs represent an agent that binds to them very strongly, specifically, and possibly irreversibly as these structures remain labeled for at least three days after INP injection. The data show that the ISS in the tumor and its periphery are more abundant than the normal vasculature that contains identifiable endothelial cells (high levels of CD31 and flat nuclei)—the traditionally accepted mode by which tumors receive nutrients and oxygen^36^. The phenomenon of tumor cell transdifferentiation could be operative here^37^. If vascular mimicry, which is associated with tumor aggressiveness and a poor prognosis^38^ supplies a large proportion of the tumor nutrient and oxygen supply, one might expect that **1**. Antiangiogenic strategies will not work well, **2**. The response to X-ray therapy will be different from normally vascularized tumors where the endothelial cells are radiation sensitive leading to a marked reduction of blood flow to the tumor and resultant hypoxia^18^, **3**. Vascular normalization strategies may not work well, **4**. Rapid tumor progression might not depend on the development of a traditional vascular blood supply, **5**. VM may play a large role in maintaining intracerebral tumor integrity, possibly through tumor nutrition.

The use of chemotherapy to treat brain tumors is complicated by limitations on drug delivery due to a number of mechanisms including the BBB and efflux pumps that eject drugs from the tumor cells. ISS/VM might form a new kind of barrier for drug delivery to the tumor—one that might involve a connective tissue matrix barrier. The INPs may circumvent the BBB by lining tumor cells directly by targeting the ISS/VM surfaces. Efflux pumps also would seem to be ineffective for INPs lining the VM/tumor cell surface. Since INPs bind the ISS/VM/tumor cell interface well, they would be expected to amplify RT dose closer to critical tumor cell organelles such as nuclei, mitochondria, cell membranes as well as the ISS/VM itself; ISS/VM targeting by nanoparticles/INPs/small molecules might serve to enhance drug delivery to intracerebral breast tumors. Since tumor stem cells have been reported to play a role in forming VM channels^39–41^, it is possible INP-RT provides a specific way to amplify RT dose to the tumor stem cell population more specifically. More experimentation is needed to explore these and other mechanisms to explain INP-RT efficacy in the 231 and PDX models—some of which are reported to be non-leaky.

## Conclusion

INPs, CD31 and Col I appear to colocalize predominantly to ISS (likely VM) found throughout the 231-tumor growing in the mouse brain—but most strongly in a rim at the tumor periphery. These structures may be important therapeutic targets. If so, it might be possible to use them for increased drug delivery, immunotherapy or oncolytic virus therapy in combination with radiation therapy.

## Supplementary Information

Supplementary Information.
