# Supplementary material for: Novel Iodine nanoparticles target vascular mimicry in intracerebral triple negative human MDA-MB-231 breast tumors
Source: Sci Rep. 2021 Jan 13;11:1203. doi: 10.1038/s41598-020-80862-5 (PMC7806637; doi:10.1038/s41598-020-80862-5)
Supplement: Supplementary file 1 — Supplementary Information. [file 41598_2020_80862_MOESM1_ESM.docx]

**Novel Iodine Nanoparticles Target Vascular Mimicry in Intracerebral Triple Negative Human MDA-MB-231 Breast Tumors**

Sharif M. Ridwan^1*^, James F. Hainfeld^2*^, Vanessa Ross^1^, Yaroslav Stanishevskiy^2^, Henry M. Smilowitz^1#*^

^1^Department of Cell Biology, University of Connecticut Health Center, 263 Farmington Avenue,

Farmington, CT 06030

^2^Nanoprobes, Inc., 95 Horseblock Road, Yaphank, NY 11980

# Corresponding Author

*Sharif M Ridwan, James F Hainfeld and Henry M. Smilowitz contributed equally to this study.

**Supplementary Data:**

**
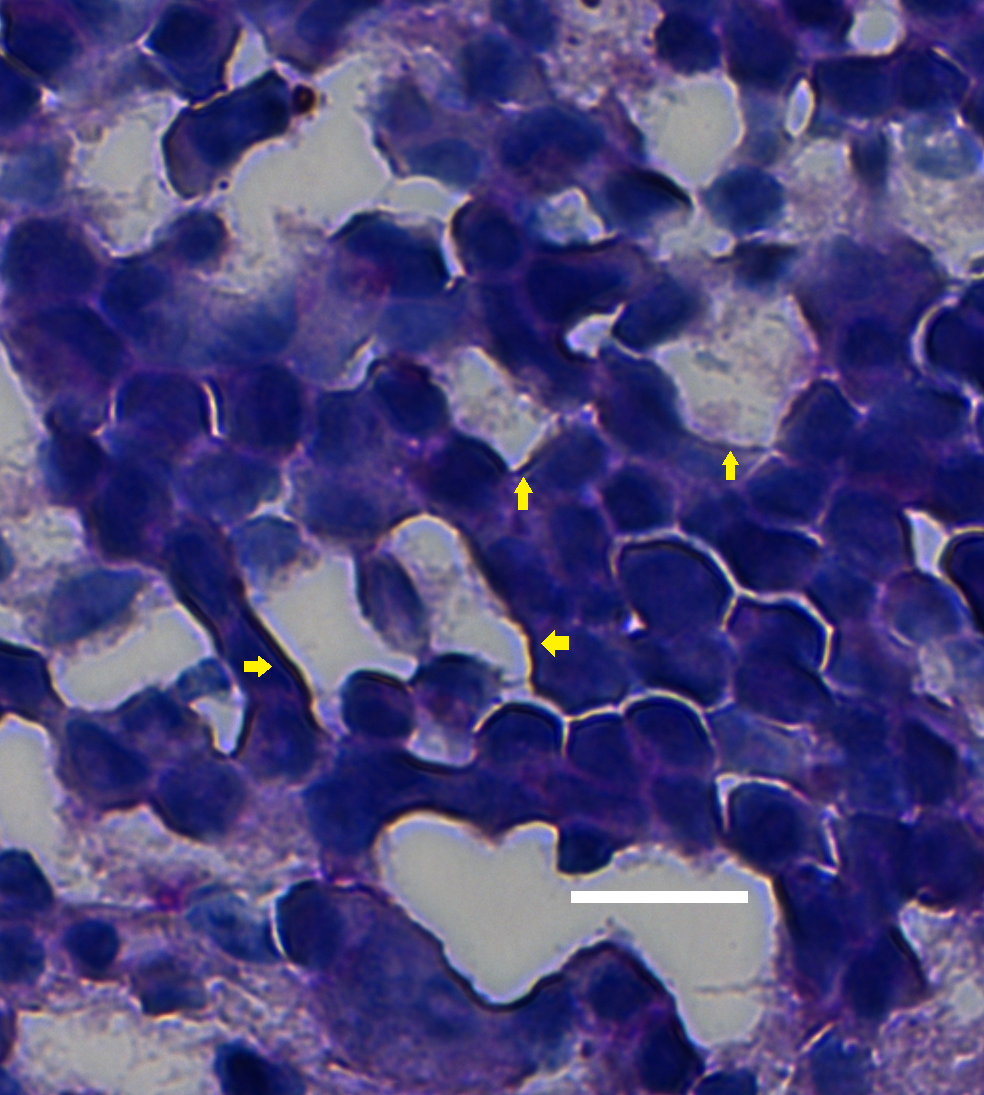
**

**Supplementary Figure 1:** PAS staining from figure 3F enlarged. **White bar = 20µm**.


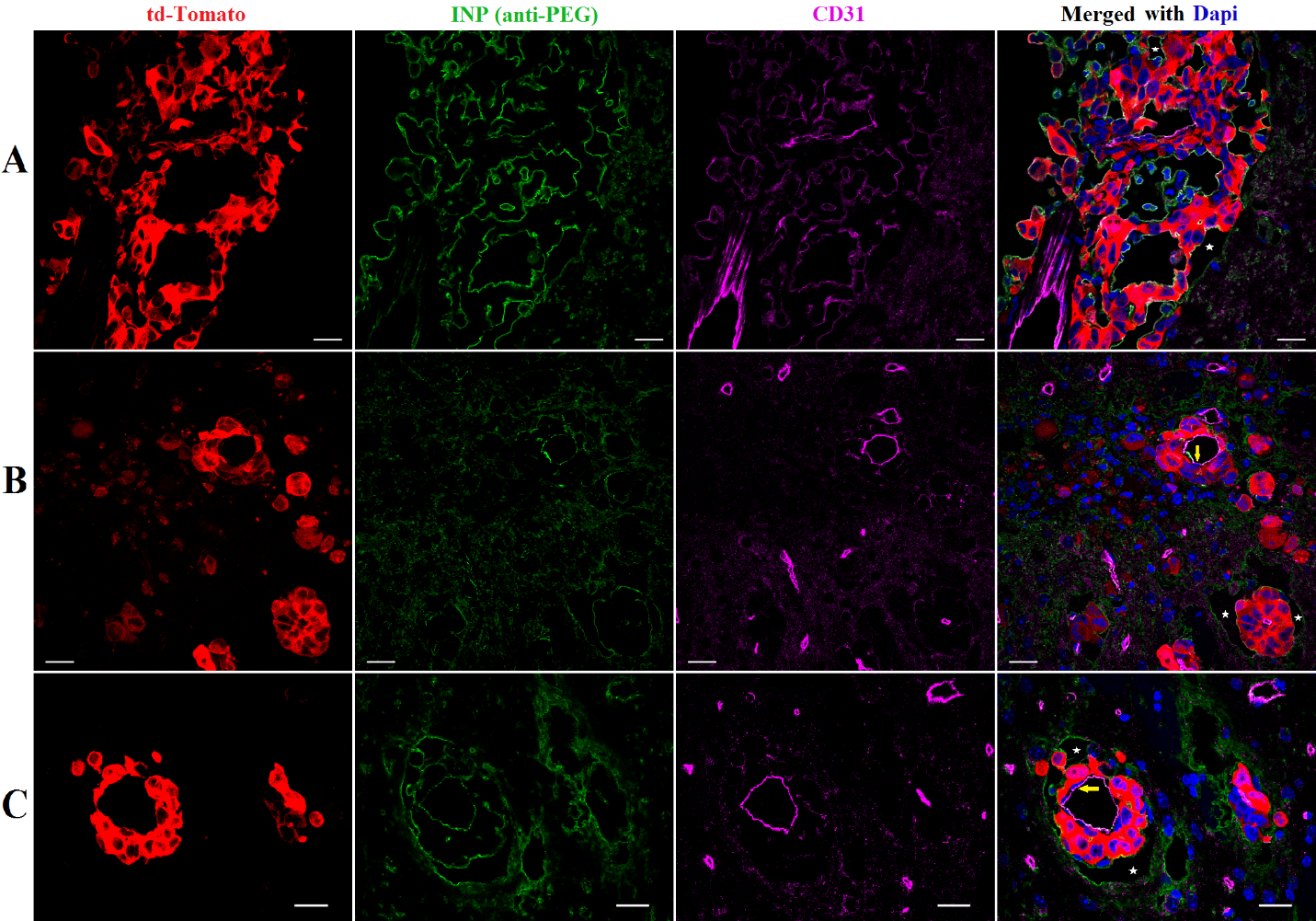


**Supplementary Figure 2: Fluorescence labeled confocal images (63x) of brain coronal section of athymic nude mouse bearing MBD-MD-231 tumors sacrificed 72 hours after iv INP injection.** INP distributions are similar to that seen 24 hours after INP injection. A. Tumor and peritumor region; B, C. Migrated tumors deep in the left cerebrum. Tumor cells, td-Tomato, red; INPs, anti-PEG, green; Endothelial stain, anti-CD31, violet; Nuclei, Dapi-Blue. Asterisks = ISS; Yellow arrows = flattened nuclei of endothelial cells. White bar = 20µm.


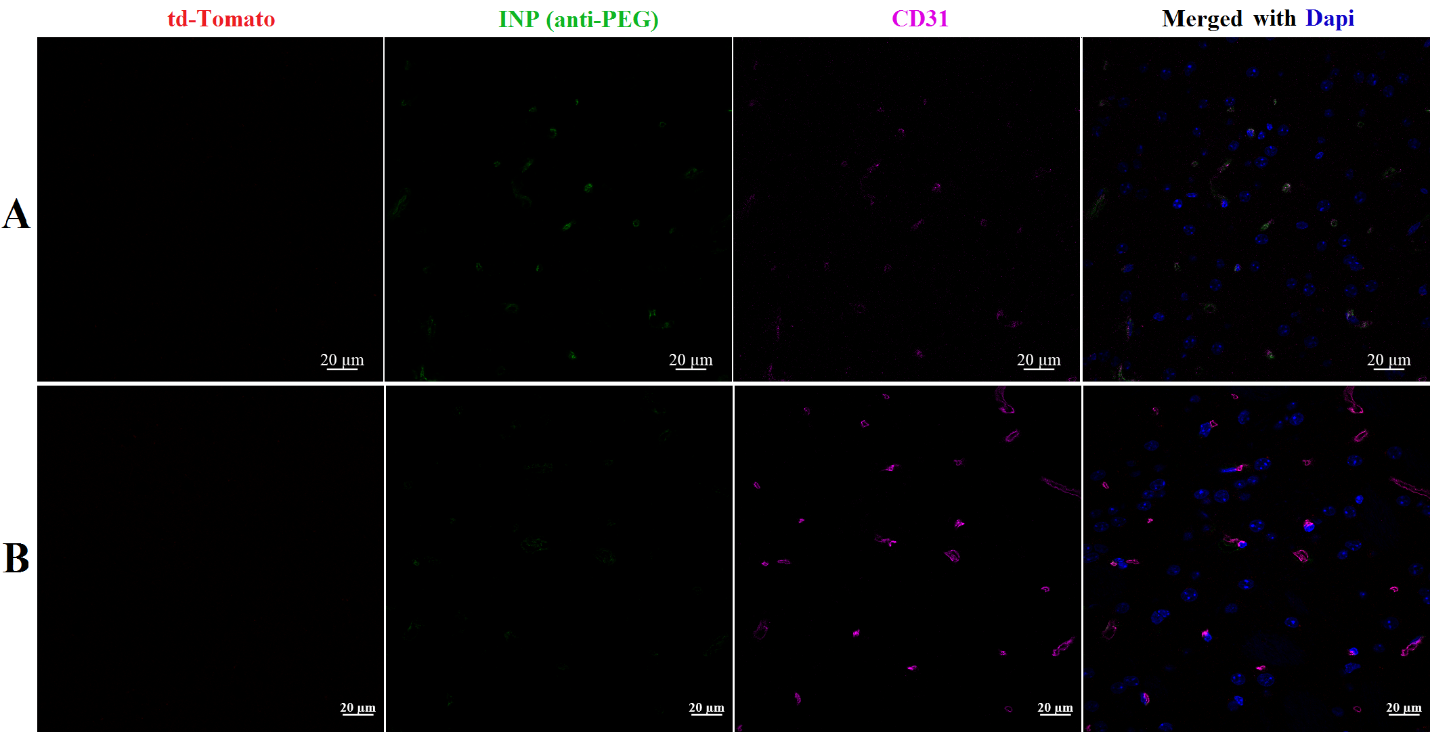


**Supplementary Figure 3:** C**onfocal (63x) images of tumor free right hemispheres of brains from mice bearing orthotopic U87 glioma perfusion-fixed 24 hours (A) and 72 hours (B) after iv INP injections (7g/kg).** Tumor cells are expressing td-Tomato, red, INPs, green, endothelial stain, anti-CD31, violet, nuclei, Dapi (blue). Very little INP staining is seen associated with anti-CD31 stain.


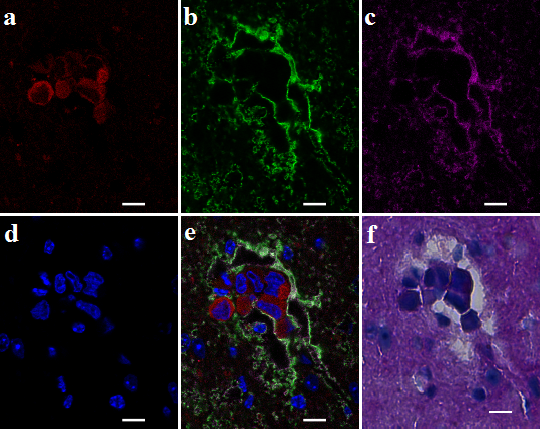


**Supplementary Figure 4: Migrating MBD-MD-231 tumor cells 24 hours after iv INP.** a. Tumor cells (td-Tomato, red); b. INPs, anti-PEG, green; c. Endothelial stain, antiCD31, violet; d, Nuclei, Dapi-Blue; e. All four colors combined, f. PAS stain. **White bar = 10 µm.**


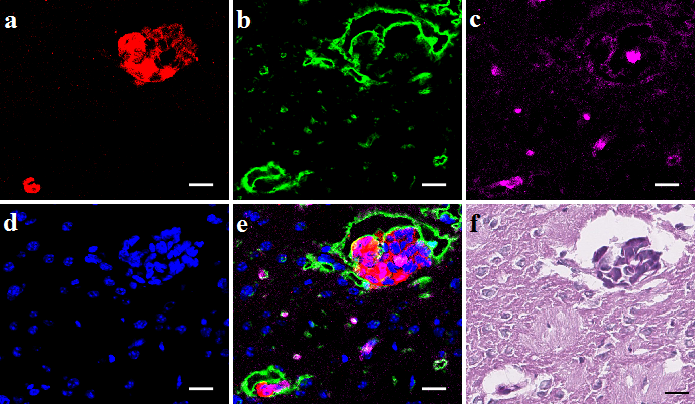


**Supplementary Figure 5: Migrating MBD-MD-231 tumor cells 24 hours after iv INP.** a. Tumor cells (td-Tomato, red); b. INPs, anti-PEG, green; c. Endothelial stain, antiCD31, violet; d, Nuclei, Dapi-Blue; e. All four colors combined, f. PAS stain. **White and black (f) bar = 10 µm.**


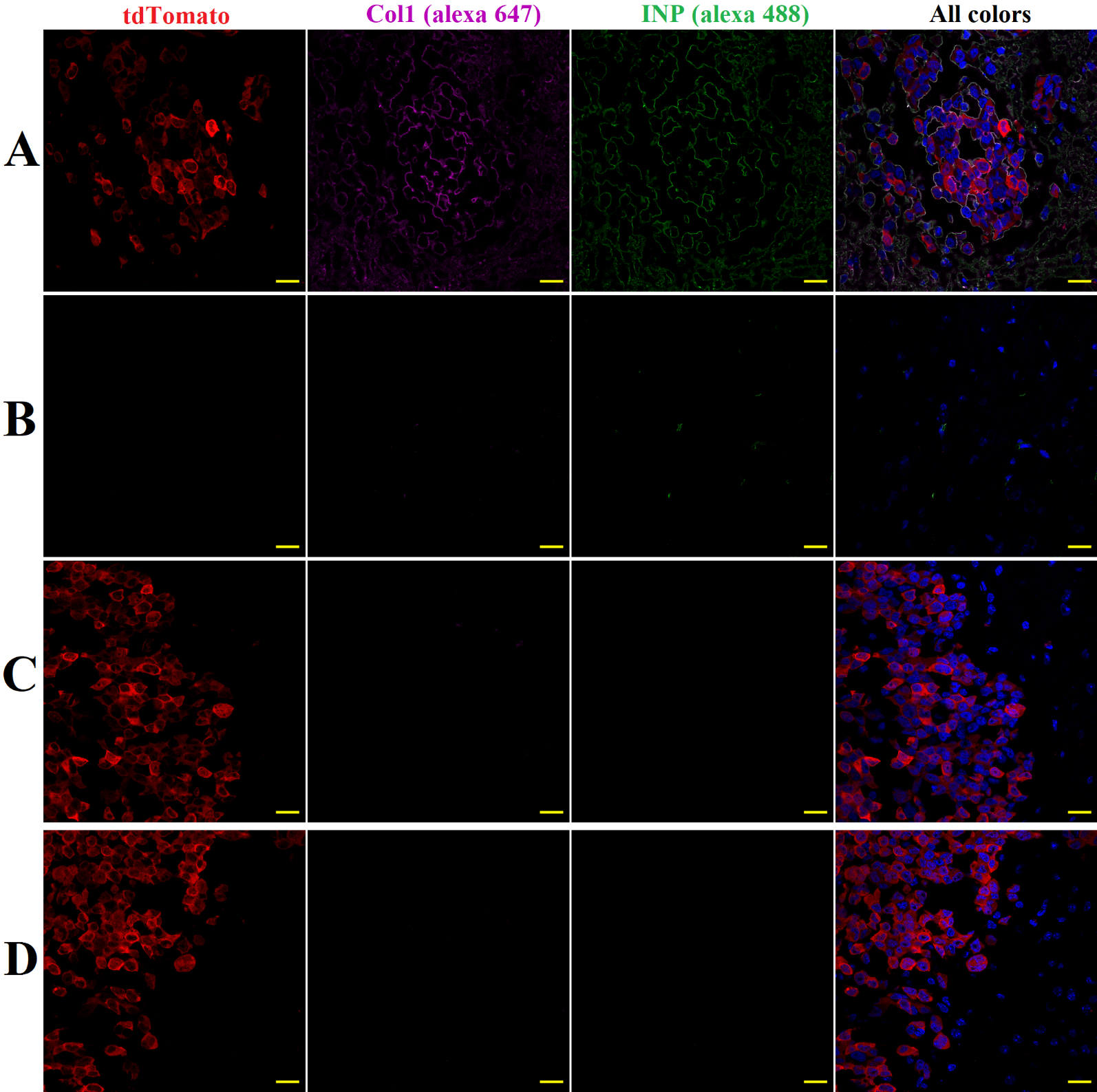


**Supplementary Figure 6:** **Confocal images (63X) of mouse brain with an advanced 231TL tumor 24 hours after iv INP injection. Fluorescent controls for Anti-Collagen I staining.** Immunofluorescence was performed as described in Methods. tDTomato = tumor cells; Green = INP; Magenta = Collagen 1; Blue = Dapi (Nuclei). A, B: Both primary and secondary ab were used. A: Tumor region left hemisphere. B: Non-tumor right hemisphere. C: No primary abs to Col1 or INP. D: No antibodies. Yellow bar = 20 µm.
